# The Neuroprotective and Anxiolytic Effects of Magnesium Sulfate on Retinal Dopaminergic Neurons in 6-OHDA-Induced Parkinsonian Rats: A Pilot Study

**DOI:** 10.3390/brainsci14090861

**Published:** 2024-08-26

**Authors:** Leyi Huang, Renxi Lin, Chunying Zhang, Shaoqing Zheng, Yiyang Wang, Zeyu Wu, Sihao Chen, Yihan Shen, Guoheng Zhang, Yuanlin Qi, Ling Lin

**Affiliations:** 1 Department of Biochemistry and Molecular Biology, Fujian Medical University, Fuzhou 350122, China; lyhuang23@163.com (L.H.); linrenxi89@fjmu.edu.cn (R.L.); zcyfjmu2020@163.com (C.Z.); millerzhang02@163.com (G.Z.); ylqi@fjmu.edu.cn (Y.Q.); 2 Key Laboratory of Brain Aging and Neurodegenerative Disease, Fujian Medical University, Fuzhou 350122, China; 3 Experimental Teaching Center of Basic Medicine, Fujian Medical University, Fuzhou 350122, China; 4 School of Clinical Medicine, Fujian Medical University, Fuzhou 350122, China; 3201007031@stu.fjmu.edu.cn (S.Z.); 3201010024@stu.fjmu.edu.cn (Y.W.); wuzeyu@stu.fjmu.edu.cn (Z.W.); chensihao@stu.fjmu.edu.cn (S.C.); 18065623660@163.com (Y.S.)

**Keywords:** Parkinson’s disease, magnesium ion, magnesium ion transporter, retina, tyrosine hydroxylase

## Abstract

This study investigates the protective effects of magnesium sulfate on dopamine neurons in the retinas of rats with 6-hydroxydopamine (6-OHDA)-induced Parkinson’s disease (PD). Rapidly progressing cognitive decline often precedes or coincides with the motor symptoms associated with PD. PD patients also frequently exhibit visual function abnormalities. However, the specific mechanisms underlying visual dysfunction in PD patients are not yet fully understood. Therefore, this study aims to investigate whether magnesium homeostasis affects dopaminergic neurons in the retina of PD rats. Thirty-six rats were divided into four groups: (1) control, (2) control with magnesium sulfate (control/MgSO_4_), (3) Parkinson’s disease (PD), and (4) Parkinson’s disease with magnesium sulfate (PD/MgSO_4_). The apomorphine-induced (APO) rotation test assessed the success of the PD models. The open-field experiment measured the rats’ anxiety levels. Tyrosine hydroxylase (TH) and glutamate levels, indicators of dopamine neuron survival, were detected using immunofluorescence staining. Protein levels of solute carrier family 41 A1 (SCL41A1), magnesium transporter 1 (MagT1), and cyclin M2 (CNNM2) in the retina were analyzed using Western blot. Results showed that, compared to the PD group, rats in the PD/MgSO_4_ group had improved psychological states and motor performance at two and four weeks post-surgery. The PD/MgSO_4_ group also exhibited significantly higher TH fluorescence intensity in the left retinas and lower glutamate fluorescence intensity than the PD group. Additional experiments indicated that the protein levels of SLC41A1, MagT1, and CNNM2 were generally higher in the retinas of the PD/MgSO_4_ group, along with an increase in retinal magnesium ion content. This suggests that magnesium sulfate may reduce glutamate levels and protect dopamine neurons in the retina. Thus, magnesium sulfate might have therapeutic potential for visual functional impairments in PD patients.

## 1. Introduction

Parkinson’s disease (PD) is one of the most prevalent neurodegenerative diseases [1]. The primary pathological features include a reduction in dopaminergic neurons in the substantia nigra-striatal pathway and the aggregation of Lewy bodies within neurons [2]. PD manifests as both motor and non-motor symptoms. Visual dysfunction is an early non-motor symptom of PD, often preceding motor symptoms, making it significant for early diagnosis and treatment [3]. Studies indicate that PD patients experience poorer vision, reduced contrast sensitivity, and impaired color discrimination compared to healthy individuals, linked to α-synuclein accumulation in the retina and a decrease in dopamine neurons [4]. Despite its clinical importance, there is currently no targeted treatment for visual dysfunction in PD.

Recently, the potential benefits of magnesium ion therapy for PD have gained attention. Magnesium ions play vital roles in many physiological processes [5]. Cellular magnesium homeostasis is primarily regulated by ion channels and magnesium transporters on cell membranes [6]. Research has shown that magnesium levels in the brain, cerebrospinal fluid, blood, urine, and hair are significantly lower in PD patients compared to healthy individuals [7,8]. Animal studies indicate that a high-magnesium diet or magnesium-containing drugs can improve motor symptoms and reduce dopaminergic neuron damage in PD rats [9,10]. Thus, magnesium homeostasis may be closely related to PD development.

Magnesium ions act as natural calcium blockers, reducing calcium influx, inhibiting reactive oxygen species production, reducing oxidative stress, and protecting dopaminergic neurons by inhibiting glutamate N-methyl-D-aspartic acid (NMDA) receptors [11,12]. Commonly used drugs, such as levodopa, pramipexole, and benztropine, primarily work by dopamine replacement, dopamine receptor agonism, and cholinergic receptor antagonism [13]. In contrast, magnesium ion treatment aims to halt disease progression by reducing oxidative stress and protecting dopaminergic neurons.

Additionally, magnesium ions help maintain retinal structural stability and slow optic nerve cell loss in glaucoma patients by inhibiting glutamate excitotoxicity [14,15]. Thus, magnesium ions might also alleviate visual dysfunction in PD patients. Magnesium homeostasis depends on magnesium ion transport proteins, and their inactivation can cause magnesium deficiency. Key magnesium ion transport proteins include cyclin M2 (CNNM2), solute carrier family 41, member 1 (SLC41A1), and human magnesium transporter 1 (MagT1) [11]. Exploring the relationship between magnesium ions, these transporters, and visual dysfunction in PD is crucial for early diagnosis and treatment.

The primary aim of this study was to investigate the protective effects of magnesium sulfate on retinal dopamine neurons in PD rats and to understand the underlying mechanisms.

## 2. Materials and Methods

### 2.1. Animals

This study was conducted strictly in accordance with the recommendations in the Guide for the Care and Use of Laboratory Animals of China. The protocol was approved by the Animal Experiment Ethics Committee of Fujian Medical University (Protocol Number: 2015-26). All surgeries were performed under pentobarbital anesthesia, and we made all efforts to minimize pain and the number of animals used by adhering to the ARRIVE guidelines.

Male-specific pathogen-free (SPF) Sprague–Dawley (SD) rats (body weight 270–300 g, 12 months of age, four rats in one cage) were purchased from the Experimental Animal Center of Fujian Medical University, Fuzhou, China. The rats were bred in an SPF environment (12 h light/dark cycle, temperature of 23 ± 2 °C, humidity of 55 ± 5%) and provided free access to food and water. Rats were acclimatized for one week before experiments.

### 2.2. Experimental Grouping

The SD rats were randomized to the 14-day group and 28-day group, and the rats in each group were randomly divided into the control group, control and magnesium supplementation (control/MgSO_4_) group, PD group, and PD and magnesium supplementation (PD/MgSO_4_) group. The minimum number of rats per experimental group is six.

### 2.3. Drug Treatment In Vivo

#### 2.3.1. 6-Hydroxydopamine (6-OHDA)

The SD rats were anesthetized by intraperitoneal injection of 2% sodium pentobarbital (40 mg/kg, vehicle: ddh_2_O) and placed in a stereotaxic instrument (Narishige, Setagaya, Japan). After the skin on the head was disinfected with 75% alcohol, an incision was made to expose bregma. 6-OHDA (8 µg/2 μL) was injected into the right MFB (medial forebrain bundle) according to a rat brain stereotaxic atlas [16] (AP: −4.4 mm, ML: −1.4 mm, DV: +8.5 mm) using a modified microinjection device. 6-OHDA was slowly injected via a glass needle (2 μL/8 min), and the glass needle was left in place for 5 min before being withdrawn (5 mm/min). The corresponding control groups were injected with 2 μL normal saline (containing 0.2% vitamin C).

#### 2.3.2. Magnesium Sulfate

Rats in the control/MgSO_4_ group and PD/MgSO_4_ group received intraperitoneal injections of magnesium sulfate (50 mg/mL, 90 mg/kg) 30 min post-surgery and subsequently on a daily basis for either 14 or 28 consecutive days.

### 2.4. Apomorphine (APO)-Induced Rotation Test

Fourteen days or twenty-eight days after 6-OHDA lesioning, the rats were injected intraperitoneally with apomorphine (0.5 mg/kg) to induce rotational behavior. Rats were considered successful PD rat models if they rotated stably toward the contralateral side for 210 rotations within 30 min (or 7 r/1 min).

### 2.5. Open-Field Test

Fourteen days/twenty-eight days after 6-OHDA lesioning, the rats were placed in the center of an open field arena (40 cm × 40 cm × 35 cm) and allowed to explore freely for 5 min. The total distance traveled, distance traveled in the central zone, and activity in the central zone were measured to assess the anxiety of the rats.

### 2.6. Immunofluorescence Staining

The rats were decapitated, and their eyeballs were removed and fixed in an ocular fixative solution (composed of glacial acetic acid, formaldehyde, anhydrous ethanol, and physiological saline) (Servicebio, Wuhan, China) for 24 h. The tissues were dehydrated, cleared in xylene, embedded in paraffin, and cut into 5 μm sections centered on the macula along the sagittal plane. After dewaxing and antigen retrieval, paraffin sections were blocked with 5% normal goat serum for 30 min and incubated overnight at 4 °C with primary antibodies [anti-tyrosine hydroxylase (TH) antibody (1:300) and anti-glutamate antibody (1:200)]. The next day, after rinsing in PBS, the sections were incubated with CY3-labeled anti-mouse secondary antibodies (1:300) for 60 min at room temperature and quenched with autofluorescence quencher for 30 min. After the nuclei were stained with DAPI, the sections were sealed with an anti-fluorescence quenching agent. Five visual fields (×400) were randomly selected for observation with a Leica SP5 laser microscope (Leica, Wetzlar, Germany), and the images were processed with LAS AF Lite software (4.0.11706 (x64)).

### 2.7. Estimation of Magnesium Ion Content in Rat Retinal Tissues

After anesthesia, the rats were decapitated, their eyes were enucleated, and the preocular segment was removed rapidly on ice. After the addition of a 9× sample volume of PBS, the tissues were ground with a tissue grinder (Servicebio, Wuhan, China). The homogenates were centrifuged for 10 min at 4000 rpm, and the supernatants were collected. Two hundred microliters of working solution were added to the wells of a 96-well plate, and the plate was incubated for 5 min at 37 °C. Then, 10 μL of double-distilled water, magnesium standard solution (1.0 mmol/L), and tissue samples were added to the blank wells, standard wells, and sample wells, respectively. The mixtures were incubated for 2 min at 37 °C. The absorbance of each sample was measured with a microplate reader at a wavelength of 550 nm, and the magnesium ion content was calculated according to the following formula:

$${\mathrm{Mg}}^{2+}(\mathrm{mmol}/L)=\frac{\mathrm{OD}(\mathrm{sample})-\mathrm{OD}(\mathrm{blank})}{\mathrm{OD}(\mathrm{standard})-\mathrm{OD}(\mathrm{blank})\;}$$

### 2.8. Western Blot Analysis of Magnesium Ion Transporter Expression in Retinal Tissue

After anesthesia, the rats were decapitated, their eyes were enucleated, and the preocular segment was removed rapidly on ice. The tissues were homogenized in RIPA buffer and centrifuged at 13,000 rpm for 10 min at 4 °C. The protein content of the supernatant was estimated using a BCA assay (Beyotime Biotechnology, Nantong, China). SDS-sample buffer was added to the remaining supernatant, and the mixture was boiled in a 100 °C water bath for 5 min. Ten percent polyacrylamide gel electrophoresis was performed to assess the expression of magnesium ion transporters. Twenty microliters (25 μg) of each protein sample and 5 μL of protein marker were added to the wells. After the proteins were separated, they were transferred to a PVDF membrane, which was then blocked in 5% skimmed milk for 2 h at room temperature. After washing with PBS, the membrane was incubated with primary antibodies (CNNM2, 1:100, Invitrogen, Waltham, MA, USA; SLC41A1, 1:100, Novus, Vancouver, BC, Canada; MagT1, 1:100, Invitrogen; GAPDH, 1:5000, Abclonal, Woburn, MA, USA) overnight at 4 °C. After washing, the membrane was incubated with secondary antibody (HRP-conjugated goat anti-rabbit, 1:2000; HRP-conjugated goat anti-mouse, 1:5000; Immunoway, Plano, TX, USA) for 2 h at room temperature. Finally, the membrane was imaged with a chemiluminescent imager (Gene), and ImageJ software was used for quantitative analysis of the blots (Table 1).

### 2.9. Statistical Analysis

All statistical analyses were performed with IBM SPSS Statistics 20, and graphs were created with GraphPad Prism 8. All results are expressed as the mean ± standard error of the mean (SEM). One-way analysis of variance (ANOVA) was used for comparisons between multiple groups, and the Wilcoxon signed-rank test was used when the variances within the groups were not homogeneous. Correlations were analyzed using Pearson correlation analysis. A *p*-value of <0.05 indicates statistical significance.

## 3. Results

### 3.1. Successful Establishment of the 6-OHDA-Induced Rat Model of PD

**Figure 1:**
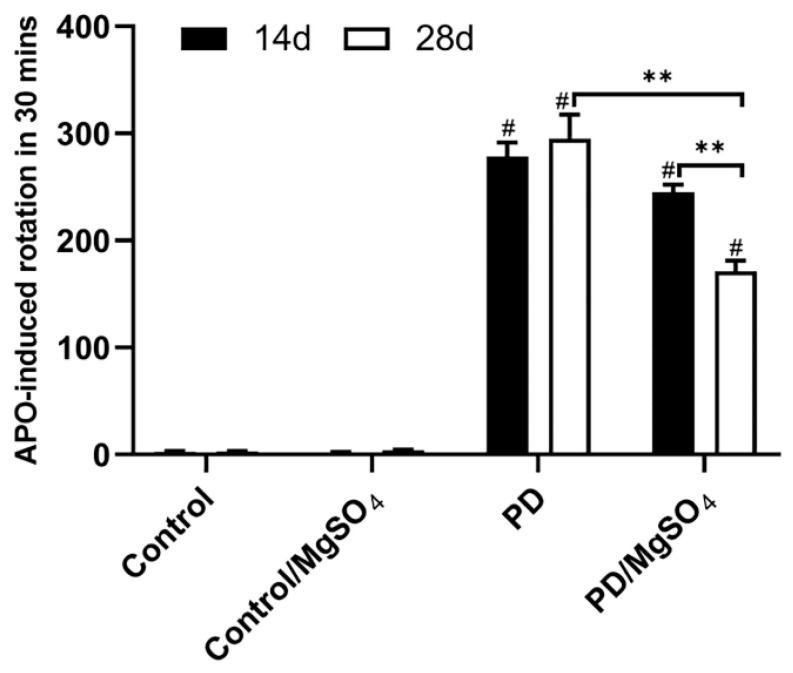
The APO-induced rotation test was performed 14 days and 28 days after surgery. # indicates the PD group vs. the control group, *p* < 0.01; ** indicates the PD/MgSO_4_ group vs. the PD group at 28 days after surgery and the PD/MgSO_4_ group at 28 days vs. the PD/MgSO_4_ group at 14 days, *p* < 0.01; N = 6.

The apomorphine-induced rotation (APO) test was used to confirm the successful establishment of the PD model and evaluate motor symptoms 14 and 28 days after the 6-OHDA injection. All PD rats met the rotation criteria of 210 rotations per 30 min or 7 rotations per minute. As shown in Figure 1, the PD groups exhibited a significant increase in rotations on days 14 and 28 compared to the control groups (*p* < 0.01). By day 28, the PD/MgSO_4_ group showed a significant decrease in rotations compared to the PD group (*p* < 0.01), indicating that sustained magnesium sulfate administration effectively delayed motor function decline in PD rats.

### 3.2. Magnesium Sulfate Improved Anxiety State and Behavior in a 6-OHDA-Induced Rat Model of PD

**Figure 2:**
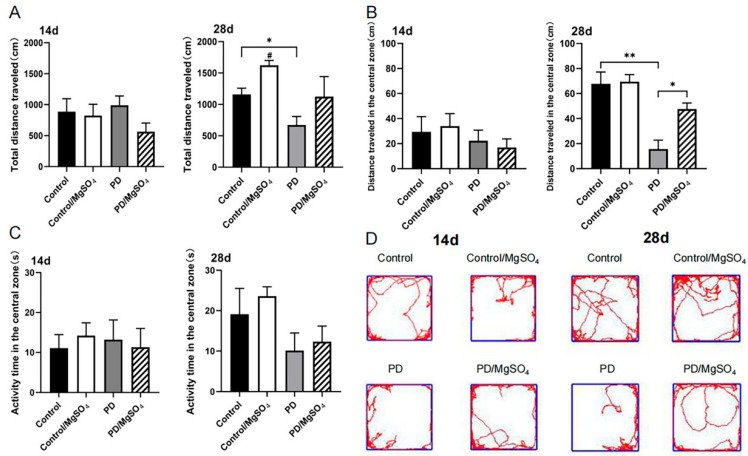
(**A**) Total distance traveled. (**B**) Distance traveled in the central zone. (**C**) Activity time in the central zone. (**D**) Activity traces. # indicates the Control/MgSO_4_ group vs. the control group at 28 days after surgery, *p* < 0.05; ** indicates the PD group vs. the control group at 28 days after surgery, *p* < 0.01; * indicates the PD group vs. the control group and the PD/MgSO_4_ group vs. the PD group at 28 days after surgery, *p* < 0.05; N = 6.

The open field test was conducted to assess motor ability and anxiety (Figure 2). We found that after 14 days of magnesium sulfate administration, the behavioral performance of the PD group of SD rats did not show significant improvement, which may be related to the efficiency of magnesium sulfate crossing the blood-brain barrier. However, compared to the control group, the PD group traveled a significantly shorter total distance and a shorter distance in the central zone 28 days post-surgery (*p* < 0.05). Magnesium sulfate injection increased the distance traveled in the central zone by the rats (*p* < 0.05). This suggests that after 28 days of magnesium sulfate administration, the locomotor activity of the PD rats significantly improved, depressive symptoms were alleviated, and their exploratory behavior further increased.

### 3.3. Magnesium Sulfate Effectively Protected Dopaminergic Neurons in Rats’ Retina

**Figure 3:**
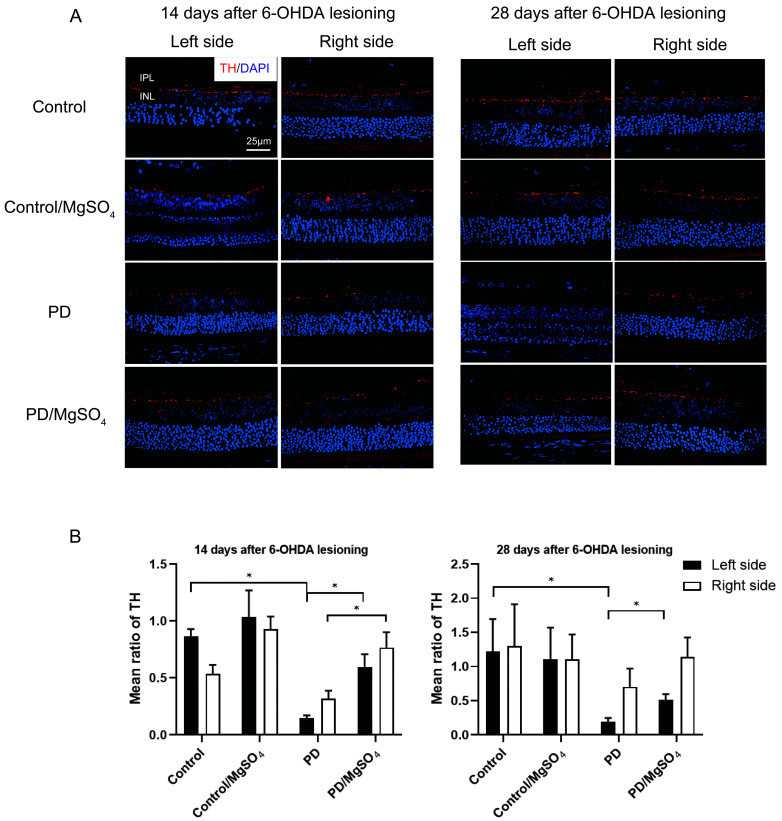
(**A**) TH fluorescence was observed under a laser confocal microscope (bar = 25 μm) (red: CY3, blue: DAPI). IPL: inner plexiform layer; INL: inner nuclear layer. (**B**) TH immunofluorescence staining intensity in rat retinal tissue. * indicates the PD group vs. the control group (left side) and the PD/MgSO_4_ group vs. the PD group (right side) at 14 days after surgery and the PD group vs. the control group (left side) and the PD/MgSO_4_ group vs. the PD group (left side) at 28 days after surgery, *p* < 0.05, N = 6.

Tyrosine Hydroxylase (TH), the enzyme critical for dopamine synthesis, was used to assess the survival of retinal dopaminergic neurons. As shown in Figure 3, 14 days post-surgery, the PD group had an 82.67% ± 0.26% (*p* < 0.05) decrease in retinal TH fluorescence intensity on the left side compared to controls, while the PD/MgSO_4_ group showed increases of 298.80% ± 74.28% and 139.82% ± 42.45% (*p* < 0.05) compared to the PD group. At 28 days, TH fluorescence intensity in the PD group decreased by 92.63% ± 2.23% compared to controls, while the PD/MgSO_4_ group had a 166.58% ± 43.71% increase compared to the PD group (*p* < 0.05).

### 3.4. Magnesium Sulfate Reduced Retinal Glutamate Content in Rats’ Retina

**Figure 4:**
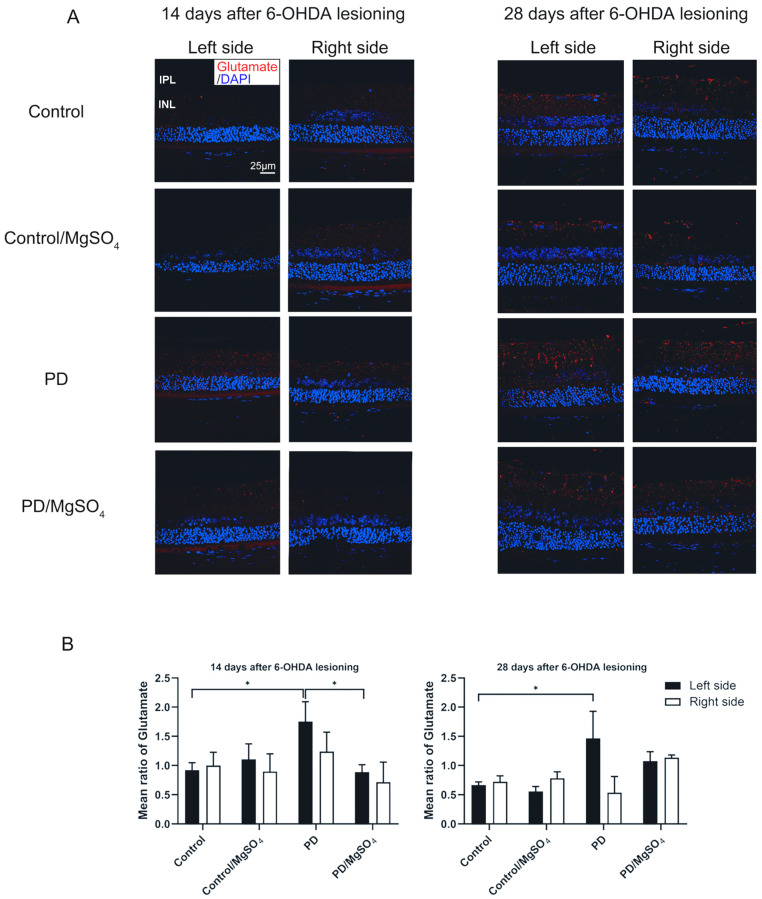
(**A**) Glutamate fluorescence was observed under a laser confocal microscope (bar = 25 μm) (red: CY3, blue: DAPI). IPL: inner plexiform layer; INL: inner nuclear layer. (**B**) Glutamate immunofluorescence staining intensity in rat retinal tissue. * indicates the PD group vs. the control group (left side) and the PD/MgSO_4_ group vs. the PD group (left side) at 14 days after surgery and the PD group vs. the control group (left side) at 28 days after surgery, *p* < 0.05; N = 6.

Glutamate accumulation and the resulting neuro excitotoxicity significantly contribute to dopaminergic neuron death. As shown in Figure 4, the PD group had a 90.22% ± 36.96% and 118.00% ± 68.66% increase in glutamate content in left retinal tissue at 14 and 28 days post-lesioning, respectively (*p* < 0.05). In contrast, the PD/MgSO_4_ group had a 49.14% ± 7.43% decrease in glutamate content at 14 days compared to the PD group (*p* < 0.05). Continuous magnesium sulfate administration effectively reduces retinal glutamate levels, protecting dopaminergic neurons.

### 3.5. Correlation between TH Fluorescence Intensity and Glutamate Fluorescence Intensity in Rat Retinal Tissue

**Figure 5:**
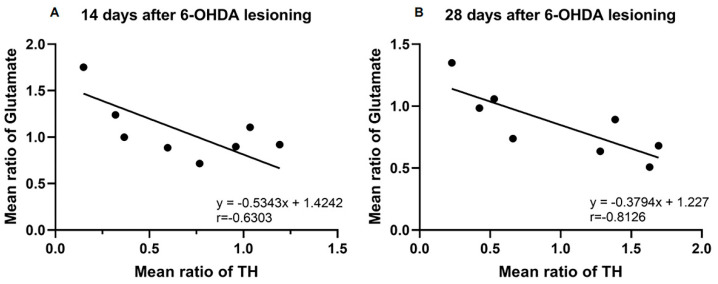
Correlation analysis of the TH fluorescence intensity with the glutamate fluorescence intensity in the rat retina at 14 days ((**A**), *p* > 0.05, N = 8) and 28 days ((**B**), *p* < 0.05, N = 8) after surgery.

The relationship between TH and glutamate fluorescence intensity in rat retinal tissue was analyzed using the Pearson correlation (Figure 5). Fourteen days post-surgery, there was no significant correlation between TH and glutamate fluorescence intensity (r = −0.6303, *p* > 0.05, N = 8). However, by day 28, there was a significant negative correlation (r = −0.8126, *p* < 0.05, N = 8).

### 3.6. Magnesium Ion Content in the Retina

**Figure 6:**
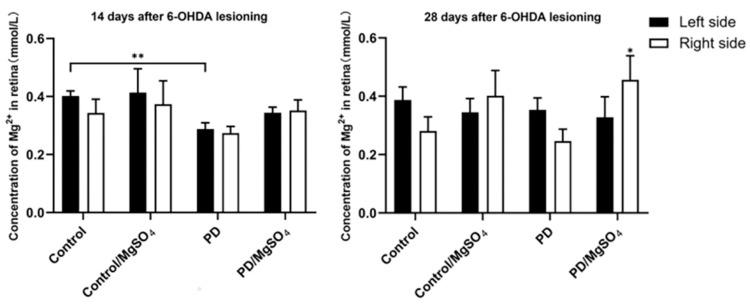
Magnesium ion content in rat retinal tissue at 14 and 28 days after surgery was determined with a magnesium ion kit. ** indicates the left side in the PD group vs. the left side in the control group at 14 days after surgery, *p* < 0.01; * indicates the right side in the PD/MgSO_4_ group vs. the right side in the PD group at 28 days after surgery, *p* < 0.05; N = 6.

As shown in Figure 6, 14 days post-surgery, the PD group had a 27.50% ± 5.00% decrease in magnesium ion content in the left retina compared to controls (*p* < 0.01). The other groups showed no significant differences. However, the PD/MgSO_4_ group showed an increasing trend in magnesium ion content compared to the PD group. At 28 days, the magnesium ion content in the right retina increased by 84.00% ± 32.00% in the PD/MgSO_4_ group compared to the PD group (*p* < 0.05). This indicates that intraperitoneal administration of magnesium sulfate can indeed increase the magnesium ion content in the retinas of PD rats to some extent. As the magnesium levels rise, the dopaminergic neurons in the retina receive a certain degree of protection.

### 3.7. Expression Levels of Magnesium Ion Transporters Detected by Western Blots

**Figure 7:**
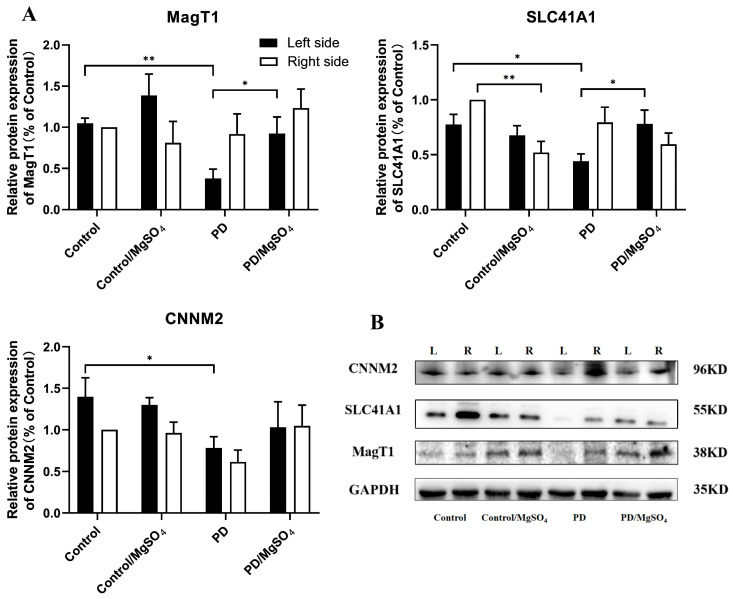
(**A**) Protein expression of the magnesium ion transporters CNNM2, SLC41A1, and MagT1 in rat retina at 14 days after surgery. (**B**) Representative Western blot. The relative expression in the right retina of the control group was set as 100%, and the relative expression for each group was calculated as (the experimental group gray value/the internal reference gray value)/(the control group gray value/the internal reference gray value). * indicates *p* < 0.05; ** indicates *p* < 0.01; N = 6.

As shown in Figure 7, 14 days post-surgery, the protein expression of magnesium ion transporters MagT1, SLC41A1, and CNNM2 in the left retina (contralateral side) was down-regulated by 63.93% ± 10.48% (*p* < 0.01), 41.03% ± 8.97%, and 44.29% ± 9.29% (*p* < 0.05), respectively, in the PD group compared to the control group. The PD/MgSO_4_ group showed upregulation of MagT1 and SLC41A1 by 144.20% ± 52.63% and 77.27% ± 29.55% (*p* < 0.05), respectively, compared to the PD group. Additionally, SLC41A1 protein expression in the right retina decreased by 48.00% ± 10.00% (*p* < 0.01) in the control/MgSO_4_ group compared to the control group.

**Figure 8:**
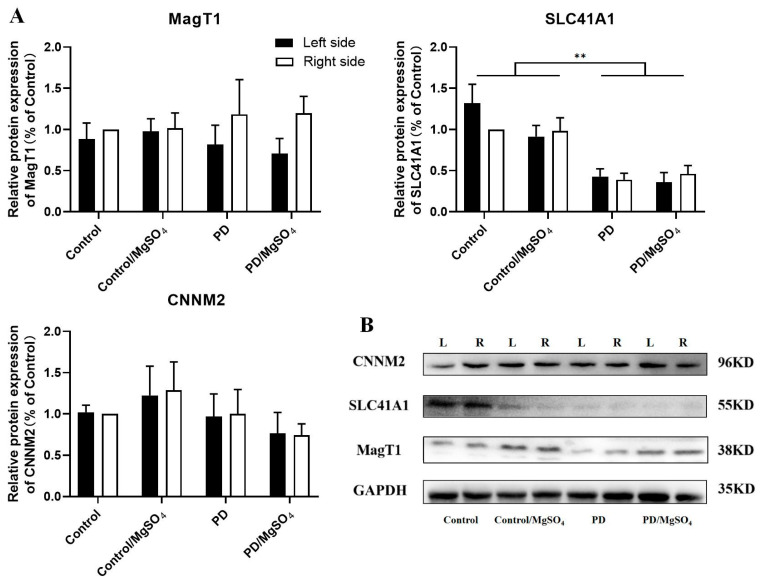
(**A**) Protein expression of the magnesium ion transporters CNNM2, SLC41A1, and MagT1 in rat retina at 28 days after surgery. (**B**) Representative Western blot. The relative expression in the right retina of the control group was set as 100%, and the relative expression for each group was calculated as (the experimental group gray value/the internal reference gray value)/(the control group gray value/the internal reference gray value). ** indicates *p* < 0.01; N = 6.

As shown in Figure 8, 28 days post-surgery, SLC41A1 protein expression in the bilateral retina was downregulated by 67.42% ± 6.82% and 61.00% ± 8.00% in the PD group and by 60.44% ± 13.19% and 53.06% ± 10.20% in the PD/MgSO_4_ group compared to the control group (*p* < 0.01). However, the protein expression levels of MagT1 and CNNM2 did not significantly differ among the groups.

## 4. Discussion

The retina is rich in dopamine. This study used a PD rat model created through unilateral 6-OHDA administration to observe the effects of magnesium sulfate on motor symptoms, retinal dopaminergic neuron survival, retinal magnesium ion content, and magnesium ion transporter expression. The aim was to explore the changes in retinal magnesium ions and transporters during PD progression and to investigate the protective effects of magnesium sulfate on retinal dopaminergic neurons.

The APO-induced rotation test is a well-accepted method to confirm successful PD modeling with unilateral 6-OHDA in rats [17]. Our previous studies showed that rats in the PD/MgSO_4_ group had significantly fewer rotations than the PD group after 28 days of continuous magnesium sulfate administration. This improvement was also noted after 14 days, suggesting that early, continuous magnesium sulfate administration can effectively enhance motor symptoms in PD rats. This finding aligns with Lin et al.’s results in human neuroblastoma cells and animal models [18,19] and supports Shindo et al.’s proposal that magnesium ions have neuroprotective effects [20].

Our study also found that PD rats were less active, more anxious, and less exploratory in the open field test. Antipova et al. [21] similarly observed motor retardation and slowness in 6-OHDA-lesioned PD rats, suggesting that PD rats exhibit psychological symptoms like anxiety alongside motor function impairment. Furthermore, continuous magnesium sulfate administration improved motor symptoms and reduced anxiety in PD rats, consistent with findings by Hajizade et al. [22] that magnesium mitigates motor and psychological symptoms.

In the retina, dopamine is synthesized by A18 cells, primarily located in the inner plexiform and inner nuclear layers [23,24]. Tyrosine Hydroxylase (TH) is a key enzyme in dopamine synthesis and indicates dopamine levels [25]. Our study found that TH fluorescence intensity in the left retina’s inner plexiform and inner nuclear layers decreased by over 80% and 90% in the PD group compared to controls at 14 and 28 days post-lesion. Magnesium sulfate supplementation significantly increased TH fluorescence intensity, indicating that retinal dopaminergic neuron loss worsened as PD progressed. This aligns with Lin et al. [18], who showed that 6-OHDA injection damages dopaminergic neurons in both the substantia nigra pars compacta and the contralateral retina, with magnesium sulfate offering protective effects [26].

Glutamate, a key neurotransmitter, accumulates excessively in PD patients due to α-synuclein, leading to excitotoxicity and dopaminergic neuron death through continuous NMDA receptor activation and magnesium ion loss [10,27]. In our study, 6-OHDA lesioning led to a significant decrease in dopaminergic neurons and an increase in glutamate in the left retina of PD rats at 14 and 28 days post-lesion. TH content is inversely correlated with glutamate levels. Zhang et al. [28] similarly found higher glutamate levels in MPTP-induced PD mice, with glutamate reduction reversing neuron degeneration. Chotibut et al. [29] showed decreased TH-positive neurons with reduced glutamate clearance, and their numbers rebounded with ceftriaxone treatment, which enhances glutamate clearance. Salvatore et al. [30] found that high glutamate in PD rats’ nigrostriatal pathway led to excessive Ca^2+^ efflux, calpain activation, and dopaminergic neuron death, which could be mitigated by inhibiting glutamate release. Our findings suggest that the relationship between glutamate and dopaminergic neurons in the retina mirrors that in the nigrostriatal pathway.

Continuous administration of magnesium sulfate for 14 or 28 days reduced retinal glutamate content and significantly improved dopaminergic neuron survival in PD rats. This supports Lambuk et al.’s [15] finding that magnesium alleviates neuron loss due to glutamate excitotoxicity in PD mice. This protective effect may stem from magnesium’s ability to increase cerebrospinal fluid magnesium levels, improving motor symptoms and slowing neuron degeneration [9]. However, some researchers note that changes in magnesium levels in brain tissues and blood in neurodegenerative disease models are minimal, indicating limited neuroprotective effects, possibly due to differences in administration routes, doses, and analysis methods [31,32].

Magnesium is believed to reduce α-synuclein aggregation in PD patients’ brains and decrease glutamate neurotoxicity by inhibiting NMDA receptors, protecting dopaminergic neurons [26,33]. In our study, magnesium ion concentration in the left retinas of PD rats decreased 14 days after 6-OHDA injection but normalized by 28 days. Sturgeon et al. [34] found lower magnesium content in the brains of PD patients compared to normal subjects, aligning with our findings. This suggests magnesium ion content changes in response to oxidative stress and neurodegeneration. However, some studies [35] found no significant difference in magnesium levels between PD patients and controls, indicating that tissue magnesium content may fluctuate less over time due to compensatory regulation.

After 6-OHDA injection into the right MFB, PD rats showed a more pronounced decrease in magnesium content in the left retina compared to the right. This aligns with Meng [36], who found that 6-OHDA significantly affects dopamine and melatonin content in the contralateral eye. This may occur because 6-OHDA disrupts the dopamine neuron membrane in the contralateral retina via the optic chiasm, causing Ca^2+^ overload and magnesium loss, leading to neuron death [37]. This is consistent with the fact that about 98% of retinal ganglion cells project to the contralateral midbrain superior colliculus [38]. It is important to note that due to experimental constraints and the limitations of the animal model, we were unable to use tracers to track the pathway of 6-OHDA and cannot entirely rule out the possibility of 6-OHDA reaching the retina through the cerebrospinal fluid. This is a limitation of this pilot study, and we will address this issue in future research to obtain more precise results.

Magnesium ion transporters regulate the intracellular magnesium homeostasis [6]. Our study found that 14 days after lesioning, MagT1, SLC41A1, and CNNM2 protein expression in the left retina was downregulated in the PD group compared to controls. However, in the magnesium sulfate-treated group, MagT1 and SLC41A1 expression increased, and CNNM2 tended to increase. Shindo et al. [39] found that CNMM2 mRNA expression decreased in an MPP+-induced PD cell model. Lin et al. [19] observed that 6-OHDA downregulates MagT1, SLC41A1, and CNNM2 in a PD cell model, and magnesium sulfate supplementation increases their expression in a PD rat model at 14 days post-lesion. Similar trends were seen for SLC41A1 in the striata of PD rats [18]. These results suggest that MagT1, SLC41A1, and CNNM2 are sensitive to 6-OHDA toxicity in the acute injury phase, and their expression in the retina is consistent with the striatum, responding to PD progression. Appropriate magnesium sulfate doses can upregulate MagT1 and CNNM2 and activate SLC41A1, increasing magnesium content and alleviating PD symptoms [40].

At 28 days post-6-OHDA injection, SLC41A1 protein expression was significantly downregulated in both retinas of the PD and PD/MgSO_4_ groups, while MagT1 and CNNM2 levels showed no significant differences among the groups. This indicates that magnesium homeostasis in rats is likely maintained by multiple transporters with different functions: MagT1 and CNNM2 mediate magnesium inflow, while SLC41A1 controls magnesium outflow [11]. As PD progresses, a balance among these transporters may develop. Downregulating SLC41A1 might slow magnesium loss, while MagT1 and TRPM7, which have similar functions, might compensate for each other [11]. The stable expression of MagT1 and CNNM2 in the PD group might be due to changes in other magnesium transporters.

## 5. Conclusions

In conclusion, this study found that magnesium sulfate protects dopamine neurons in the retinas of PD rats. This protection may be due to magnesium sulfate’s regulation of magnesium ion transporters, modulating retinal magnesium levels, reducing glutamate accumulation, and protecting dopaminergic neurons. Magnesium sulfate shows potential clinical value for treating visual dysfunction in PD patients. However, further research is needed to explore this in depth.

## Figures and Tables

**Table 1:** The catalog numbers for the antibodies.

Antibody	The Catalog Number
SCL41A1	NBP1-59693
MagT1	PA5-44929
CNNM2	PA5-43015
GAPDH	AC002
TH	MA1-24654

## Data Availability

The original contributions presented in the study are included in the article; further inquiries can be directed to the corresponding author.
